# Hylleraas-Configuration Interaction (Hy-CI) Non-Relativistic Energies for the 3 ${}^{1}S$, 4 ${}^{1}S$, 5 ${}^{1}S$, 6 ${}^{1}S$, and 7 ${}^{1}S$ Excited States of the Beryllium Atom

**DOI:** 10.6028/jres.125.006

**Published:** 2020-02-05

**Authors:** James S. Sims

**Affiliations:** 1 National Institute of Standards and Technology, Gaithersburg, MD 20899, USA

**Keywords:** beryllium atom excited states, Hylleraas-CI, Hy-CI, non-relativistic energies

## Abstract

In a previous work Sims and Hagstrom [*J Chem Phys *140,224312(2014)] reported Hylleraas-configuration interaction (Hy-CI) method variational calculations for the ^1^*S *ground states of the beryllium isoelectronic sequence with an estimated accuracy of 10 to 20 nanohartrees (nHa). In this work the calculations have been extended to the five higher states of the neutral beryllium atom, 3 ^1^*S*, 4 ^1^*S*, 5 ^1^*S*, 6 ^1^*S*, and 7 ^1^*S*. The best non-relativistic energies obtained for these states are -14.4182 4034 6, -14.3700 8789 0, -14.3515 1167 6,-14.3424 0357 8, and -14.3372 6649 96 Ha, respectively. The 6 ^1^*S *result is superior to the known reference energy for that state, while for the 7 ^1^*S *state there is no other comparable calculation.

## 1 Introduction

Variational methods based on explicitly correlated wave functions are known to give the most accurate upper bounds to energy states, and hence the inclusion of terms containing the interelectronic distance $r_{ij}$ in the wave function has become increasingly common, at least for few-electron atomic systems (*N* ≤ 4) (so common, in fact, that a book dealing entirely with explicitly correlated wave functions has been produced [1]). These wave functions, which are commonly referred to now as Hylleraas (Hy), follow the landmark calculation of Hylleraas [2] by employing powers of the interelectronic distance in the wave function. The Hylleraas-configuration interaction (Hy-CI) technique (developed by us [3] and also independently by Woznicki [4]) differs from the traditional Hy development by employing at most a single, linear $r_{ij}$ factor with traditional configuration interaction orbital bases. While the work of Hylleraas demonstrated that two-electron atoms could be calculated accurately (for that time) with powers of *r*_12_, it was Handy [5] who demonstrated that linear terms alone were sufficient for high accuracy for He. Hy-CI in its current incantation utilizes only linear terms in $r_{ij}$, and so $r_{12}/f_{12}$ [1, 6, 7] methods are related to Hy-CI but outside the scope of this study (but see Ruiz [8] for a discussion of CI-*r*_12_ and a comparison with Hy-CI in the two-electron He atom case). Recently, Nakatsuji and Nakashima introduced Hy-CI into their free-complement chemical formula theory (FC-CFT) [9, 10], and while their study is not nearly as extensive as the current study, this is an interesting new development. In contrast to Hy and Hy-CI wave functions, the other common explicitly correlated wave function, the explicitly correlated Gaussian (ECG) [1] wave function, has the $r_{ij}$ correlation appear as Gaussian exponentials.

As a consequence of its formalism with an at most single $r_{ij}$ factor per term, Hy-CI is unique (among the Hy methods) in that the mathematical problems for *N >* 4 can be reduced to problems with four electrons. Therefore, beryllium, with its four electrons and strong mixing of 1*s*^2^ 2*s*^2^ and 1*s*^2^ 2*p*^2^ configurations, is ideal for a test of the Hy-CI formalism. In paper I of this series, Hy-CI calculations were carried out on the ^1^*S* ground states of the Be isoelectronic series from *Z* = 4 through 113 [11]. Li^−^ (with *Z* = 3) has a decidedly different electronic structure from the other members of the sequence, and so it was not until the second paper in this series that a large, comparable calculation for the Li^−^ ground state was completed, and the results of that calculation were discussed [12]. What distinguishes Li^−^ from other members of the sequence is its negative charge, which makes for a much more diffuse electronic charge distribution than ^1^*S* ground states of the other members of the Be isoelectronic series. Specifically, the ground ^1^*S* state of Li^−^ is the same type of problem as the first excited state of Be; it is like Be(2*s*3*s*)^1^1 The successive Be ^1^*S* excited states 3 ^1^*S*, 4 ^1^*S*, *etc*., are referred to herein also as Be(2*s*3*s*), Be(2*s*4*s*), *etc*., not Be(2*s*2*s*). This work continues the work begun with Li^−^ in examining how well Hy-CI can treat states successively more diffuse than the ground state by examining successively higher states of beryllium of ^1^*S* symmetry.

A comprehensive review of the earlier non-relativistic, infinite nuclear mass calculations on the Li anion can be found in the work of King [13]. More recently, three ECG calculations have been reported [14–16], the last of which is the most extensive calculation and the best to date. The recent calculations of Hussein [17] are notable for their exploration of large-scale CI for ground and core excited states of the anion. A standard Hy calculation of more limited scope is of note for its relatively compact basis sets and its study of how well the basis sets employed can describe the more diffuse region of configuration space for this species [18]. For Be, Adamowicz and co-workers [19] reported ECG results for the five lowest ^1^*S* states of the Be atom, i.e., for ground and 2*s* → *ns* (*n* = 3-6) Rydberg excited states^2^2 Nakatsuji and Nakashima ([10]) also studied ground and 2*s*→*ns* (*n* = 3-6) Rydberg excited states of Be.. In two recent papers ([20, 21]), Adamowicz and co-workers extended these calculations to include the lowest ten ^1^*P* Rydberg states and re-examined the ground and 2*s* → *ns* (*n* = 3-5) Rydberg excited states. In this work, we treat the same 2*s* → *ns* (*n* = 3-6) Rydberg excited states and then use that methodology to extend the calculations to include the 7*s* Rydberg excited state of Be.

## 2 Variational Calculations

For *N_e_* electrons, the total non-relativistic, stationary-point-nucleus energy *E_NR_* is defined as the exact solution (eigenvalue) of the time-independent, non-relativistic Schrodinger equation


$$H_{NR}\Psi (r_{1},r_{2},...r_{N_{e}})=E_{NR}\Psi (r_{1},r_{2},...r_{N_{e}}),$$ (1)

where the Hamiltonian *H_NR_* is defined as (in atomic units)


$$H_{NR}=\sum_{i=1}^{N_{e}}H_{i}+\sum_{i<j}^{N_{e}}r_{ij}^{-1}.$$ (2)

Here, *H_i_* = *T_i_* + *V_i_*, *H_i_* is a one-electron operator (electron *i*) consisting of a kinetic energy part, *T_i_* = − ^1^ ∇^2^, and a nuclear attraction part, *V_i_* = −*Z/r_i_*. *N_e_* denotes the number of electrons, and *Z* is the 2 *^I^* corresponding nuclear charge.


$$\Psi =\sum_{K=1}^{N}C_{K}\Phi_{K},$$ (3)

where


$$\Phi_{K}=\Lambda (r_{ij}^{\nu_{K}}\prod_{s=1}^{4}\left\{\phi_{K_{s}}(r_{s})\right\}\Theta_{K})=O_{as}O_{L,M_{L}}O_{S,M_{S}}(r_{ij}^{\nu_{K}}\prod_{s=1}^{4}\left\{\phi_{K_{s}}(r_{s})\right\}\Theta_{K})$$ (4)

denotes the *K*th antisymmetrized spin and angular momentum projected configuration state function (CSF). *O_L,M__L_* and *O_S,M__S_* are idempotent orbital and spin angular momentum projection operators of the Lo¨wdin type [22] for a state of total quantum numbers *L, M_L_, S*, and *M_S_* (Russell–Saunders LS coupling is assumed). In practice, it is sufficient to take *ν_K_* equal to 0 or 1, with *ν_K_* = 0 being the CI case. Θ*_K_* is a primitive spin product function for term *K*, and *φ_Ks_* (**r_s_**) represents the *s*th basis orbital in the *K*th term.

The basis orbitals are un-normalized Slater-type orbitals (STOs), *φ* (**r**), which are defined as


$$\phi_{i}(r)=r^{n_{i}-1}{\text{e}}^{\text{-}\alpha_{\text{i}}\text{r}}{\text{Y}}_{{\text{l}}_{\text{i}}}^{{\text{m}}_{\text{i}}}(\theta ,\phi ),$$ (5)

where *Y^m^*(*θ, φ*) is a normalized spherical harmonic in the Condon and Shortley phase convention [23]. *O_as_* is the idempotent antisymmetry projection operator. For four-electron singlet states, there exist two linearly independent primitive spin functions, Θ_1_ = *αβαβ* and Θ_2_ = *ααββ* . Cencek and Rychlewski [24] have given the general proof that only one primitive spin function is needed to ensure convergence of eigenvalues to the exact root of the Hamiltonian. Here, the Θ_1_ spin function is used.

The coefficients *C_K_* in Eq.(3) are found in this work by solving the generalized eigenvalue problem


HC=ESC, (6)

$$(H-E_{0}S)C=(E-E_{0})SC,$$ (7)

$$C=(E-E_{0}){\left(H-E_{0}S\right)}^{-1}SC,$$ (8)

where the matrix elements are given by *$H_{KL}=<\Phi_{K}|H\Phi_{L}>$*and $S_{KL}=<\Phi_{K}\Phi_{L}>$, the Hamiltonian *H* is given by Eq.(2), and *E*_0_ is some starting approximation for the eigenvalue *E* of interest.

(Shifted) inverse iteration [25] is the application of the power method [26] to **A**^−1^ = (**H** − *E*_0_**S**)^−1^ in the solution process used for solving for **C** in Eq.(8) and leads to the iteration formula


$$v_{k+1}=A^{-1}Sv_{k},\;k\;=0,1,2,...$$ (9)

with a convergence criterion for *E*, computed from


$$E_{k}=E_{0}+\frac{v_{k+1}\cdot Sv_{k}}{v_{k+1}\cdot Sv_{k+1}},\;k\;=0,1,2,...$$ (10)

which converges rapidly to *E* provided that the trial *E*_0_ is sufficiently close to the desired eigenvalue, which turned out to be five digit accuracy in these calculations. In addition to a suitable *E*_0_, shifted inverse iteration requires a starting vector **v**_0_ (which was usually taken to be a vector of all 1*s*) and an efficient factorization for **A**^−1^ in Eq.(9), which, for this calculation, is


$$A^{-1}=L^{T}D^{-1}L,$$ (11)

where **D** is a diagonal matrix, and **L** is the implicit representation of the lower triangular matrix **L** in terms of Gaussian transformations [27]. Quadruple precision was used in this work, and the Message Passing Interface (MPI) Standard [28] was used to parallelize the code.

## 3 Methodology

Excited states have a more diffuse electronic charge distribution than the ground state. Atomic anions also have a more diffuse electronic charge distribution than the neutral atom of the same charge. Hence, the first excited ^1^*S* state of Be, Be(2*s*3*s*), is the same type of problem as the Li^−^*S* ground ^1^*S* state previously treated [12] and can be treated using the same basic expansion philosophy as was used previously for Be(2*s*2*s*) [11] as modified for Li^−^ [12]. This expansion philosophy is one in which the bulk of the computing involves development of a base expansion (which we refer to as the CORE), the purpose of which is to define the STO basis and the CSF basis for the most important term types, and most importantly to calculate STO orbital exponents with a reasonable amount of effort. Our first attempt at a core for Be(2*s*3*s*) is listed in Table 1 and consists of 25 blocks (9803 terms) and gives a reasonably good energy (approximately 28 *µ*Ha accuracy) for this expansion size. Table 1 and other tables of Hy-CI expansions list energy results (column 5) for various expansion lengths, *N_tot_*, shown in column 4. Column 2 lists the basis orbitals that are used to generate the CSFs (terms) for each block type in the order electron 1 (*α* spin), electron 2 (*β* spin), electron 3 (*α* spin), electron 4 (*β* spin). For example, in the first line 1:9*s_K_* for the first electron means the basis orbitals are 1*s_K_* through 9*s_K_* orbitals (where *K* indicates an orbital exponent appropriate for a *K* shell electron). Products of four orbital types are built up by taking one pair of orbital types from the *K*-shell set and the other pair from the *L*-shell set. All the orbital promotions are within the shell, not between shells. This leaves out a substantial number of CSFs that turn out to be of no importance in this work. The number of CSFs in a block can be computed from the listed basis orbitals and the value of *r*-sum, which is the sum of the powers of *r* for each Hartree orbital product of four basis orbitals [11, 29]. Column 3 gives the number of CSFs added for the block or blocks shown in column 2. In Table 1 and elsewhere, ∆*E* is the energy improvement for the blocks shown in column 1 and is shown in column 6. In Table 1 and elsewhere, for *pppp*, there are degenerate *S* states listed. One of these states is (10, 10, 10, 10), where only the *l* and *m* orbital numbers are listed. For configurations with degeneracies, *m* = 0, unless otherwise noted. In this case, both the (10, 10, 10, 10) and the (11, 11, 1-1, 1-1) configurations contribute substantially and hence are included in the CORE. The term types are what one would expect from adding correlation to a typical closed *K* shell Be CI expansion using an *s, p* STO basis.

**Table 1:**
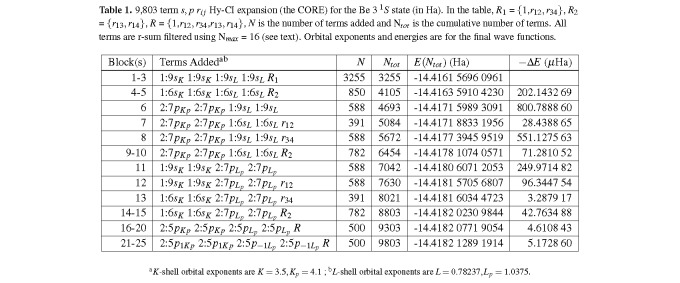
9,803 term *s, p r_i j_* Hy-CI expansion (the CORE) for the Be 3 ^1^*S* state (in Ha). In the table, *R*_1_ = *{*1,*r*_12_, *r*_34_*}*, *R*_2_= *{r*_13_, *r*_14_*}*, *R* = *{*1,*r*_12_, *r*_34_,*r*_13_, *r*_14_*}*, *N* is the number of terms added and N*_tot_* is the cumulative number of terms. All terms are r-sum filtered using N*_max_* = 16 (see text). Orbital exponents and energies are for the final wave functions.

In Table 1 and elsewhere only the orbital type for each electron is shown, and terms of each orbital type are combined with 1-5 of the five different *r_i j_* types as noted. The terms in the CORE are what one would expect from adding explicit correlation to a typical Be expansion (cf. the Be(2*s*2*s*) expansion in [11]) using an *s, p r_i j_* basis. Starting from a 10 block *s_K_s_K_s_L_s_L_*× R (*R* = {1,*r*_12_, *r*_34_, *r*_13_, *r*_14_}) + *s_K__p_ s_K__p_ s_L_s_L_*× R pre-CORE, *L* = *L_s_* and *K* = *K_s_* are optimized to self-consistency, and then *p_Kp_ p_Kp_s_L_s_L_*× R blocks are added, and K*_p_* is optimized. This unsplit *L*-shell representation, with its approximately 28 *µ*Ha accuracy, turned out to be inferior to a more natural representation for a pair of non-identical electrons in the *L*-shell. Starting from the unsplit *L*-shell (*e.g.*, 1:m*s* 1:m*s*), as in Li^−^, *L* was split symmetrically into inner and outer electrons *L*_1_ and *L*_2_ (*e.g.* 1:m*s_L_*_1_ 1:m*s_L_*_2_, where *L*_1_ and *L*_2_ are different *L*-shell orbital exponents), more representative of an excited Be(2*s*3*s*) state, and then *L*_1_ and *L*_2_ were varied. The split *L*s can be thought of as a symmetrical split of *L* into a higher inner *L* orbital exponent (less diffuse) and a lower *L* outer orbital exponent (more diffuse). In Table 2, this split shell 25 block (15,957 terms) CORE can be thought of as a base “state” of five decimal place accuracy using a correlated *s, p* STO basis, which is then augmented with enough correlated *s, p, d, f* basis blocks to achieve six decimal place accuracy. For Be(2*s*2*s*) [11], Li^−^ [12], and Be(2*s*3*s*), six decimal place accuracy is achieved with approximately 20,000 Hy-CI CSFs. For the more diffuse Li^−^ and the Be(2*sns*) states, the split shell representation ultimately gives a better energy with fewer terms in the expansion. There was considerable experimentation to determine both the size of the inner and outer pair *s* and *p* orbital exponents as well as their optimization. Note that the *L*_1_ and *L*_2_ exponents are for a 2*s*3*s* excited state, with the 3*s* orbital tending to a hydrogenic *L_s_* orbital with an exponent of -2.0/3 ≈ -0.67, where 2.0 is a guess for the effective nuclear charge (Z*_e f f_*) that the 3*s* electron sees. Similar considerations are valid for the *L*_3_ and *L*_4_ pair for the *L_p_* orbitals.

Tables 2 and A1 (Appendix) give an overall picture of the Be(2s3s) ^1^*S* energy convergence with an 82,807 term expansion using an *s, p, d, f r_i j_* STO^3^3 The STOs (Slater-type orbitals) we use were defined fully in Ref. [30]. An *s *STO has *l *= 0, a *p *STO has *l *= 1, a *d *STO has *l *= 2, *etc.* basis for the CSFs. The expansion beyond the CORE was obtained by expanding the basis to *s, p, d, f r_i j_* in a series of steps. The *spps*, *spsp*, and *ddss*× R additions to the CORE were tested, keeping blocks that contributed ≥ 1 *µ*Ha, resulting in a 40 block, 21,187 CSF expansion utilized for the second orbital optimization. This expansion was used to systematically try adding *ssdd*, *sdds*, and *sdsd* and then different *sppd* + permutation blocks, keeping all blocks that contributed ≥ 0.5 *µ*Ha to form a 76 block, 33,596 CSF “expanded CORE” utilized for the final orbital optimization. This “expanded CORE” was then expanded further by adding *f f ss*^4^4 Unless otherwise noted, the blocks are correlated., *ss f f*, *s f f s*, and selecting *s f f s*, *s f s f*, *ppdd*, *p*_1_ *p*_1_*d*_−1_*d*_−1_, *ddpp*, *d*_1_*d*_1_ *p*_−1_ *p*_−1_, *sppd*, and *s_L_*_1_*s_L_*_2_ blocks to form a 113 block, 42,139 term expansion of 0.25 *µ*Ha accuracy which formed the basis for final adjustments. A cutoff of 0.1 *µ*Ha was used in forming this expansion as well as previous experience, including the use of the Legendre expansion of possible Hy-CI term types to try [29].

The first set of adjustments^5^5 For details, see Table A1 of the Appendix. included *r*-sum and other adjustments to the CORE (*r*-sum, the sum of the powers of *r* for the four orbitals in a term, has to be ≤ 16 by default), starting with the first five blocks. The idea here is to look at the biggest energy contributors for the biggest adjustments to the 42,139 term energy, and this is done by allowing for higher powers of *r* to be included in the orbital products. Blocks 114-137 contain these adjustments where the added CSFs are obtained by taking the difference between the block listed and the block for which the block number is the number following the trailing “−#”^6^6 A process we refer to in Sec. IV as “via the difference tool”.. Doing this for all blocks in the CORE improves the result by 0.197 µHa and results in a 137 block, 62,566 term expansion accurate to 0.1 *µ*Ha (seven decimal places).

The post-CORE analysis is presented next. Up to this point, the cutoff for including blocks in the expansion has been 0.025 *µ*Ha. After lowering the cutoff to 0.4 nanohartrees (nHa), next add *pssp*+*psps* to this 137 block, 62,566 term expansion, and then add *dssd*+*dsds* and *dpdp* to form a 157 block, 70,084 term expansion. Then, test *dppd*, *pdpd*, and *pddp*. In each case (except no *r*_12_ block for *pdpd*) KKLL(1+*r*_12_) + K_1_K_1_L_−1_L_−1_ (CI block) passes the - 0.4 nHa test to give a 163 block, 71,590 term expansion with seven decimal place accuracy. The next step is to add in the remaining *sppd* and permutation contributing blocks, where keeping only blocks that contribute *>* 0.4 nHa leads to a 181 block, 77,092 term expansion. Then, *dddd, ddsd, ddds, pp f f, f f pp*, *f ss f*, and *f s f s* blocks that contribute more than the final 0.25 nHa cutoff are added. Finally, all *spd f* and permutation blocks and *s_K_*_1_ *s_K_*_1_ *s_L_*_1_ *s_L_*_2_ blocks contributing more than that cutoff are considered, and only blocks that contribute *>* 0.25 nHa are kept, which leads to our final 82,807 block, 203 block expansion with eight decimal place accuracy, given in Table 2 (overall picture) and Table A1 of the Appendix (full expansion).

In arriving at the final wave function, all possible blocks of *s, p, d* type were tried, keeping all blocks that contributed *>* 0.25 nHa. For *s, p, d, f* types, most blocks were tried, and the ones not tried were deemed unimportant at this level.

Final orbital optimization was done at the *N* = 35,678 level (33,596 term expansion in Table A1 [Appendix] plus *f f ss* + *ss f f* blocks). Due to program limitations, it was not possible to give every orbital type its own orbital exponent.

For estimating the convergence error, a record was kept of all blocks dropped and their energy contributions. For *s, p, d* orbitals, for which all blocks were tested, the sum of all the dropped blocks was 6.502 nHa. These contributions will be less sensitive when added to the final expansion than they were earlier on. Similar considerations apply for *s, p, d, f*, where there are many more blocks, and the sum of all dropped blocks adds up to 15 nHa, for a total of 22 nHa. To estimate how much less these contributions would be if added at the end of the expansion, we tested a few of them, and the contributions fell off by a factor of 2 to 3. This gives an overall estimate of convergence in our final expansion to 7-12 Ha accuracy. There is also an error arising from an inadequate STO basis. To obtain an estimate of this error, we did the following: One extra STO of each STO type was added, with separate calculations for each type and *r*-sum rule. Using only the most important CSF blocks (certainly only those in the CORE) added an additional error estimate of about 5 nHa, for an overall estimate of eight decimal place accuracy for the 3 ^1^*S* (Be[2*s*3*s*]) state. Upon examining the energy convergence in Table A1 (Appendix) and comparing it with the ground state [11], one can see that the ground state converges faster than the first excited state. This can be expected, since the excited-state wave function is more difficult to describe than the ground-state wave function due to a radial hole [31].

**Table 2:**
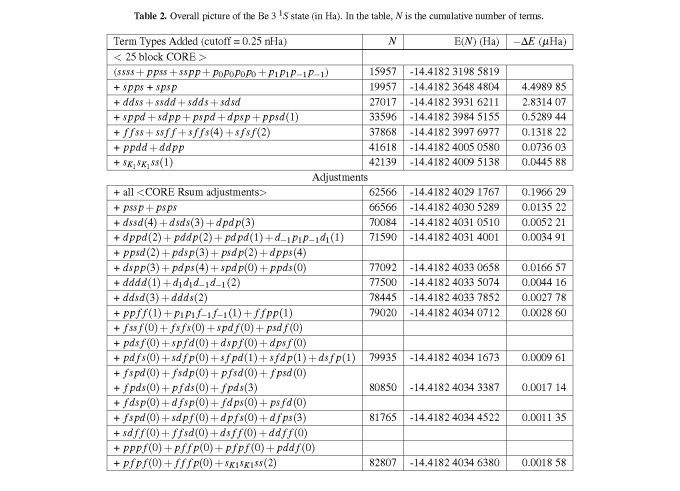
Overall picture of the Be 3 ^1^*S* state (in Ha). In the table, *N* is the cumulative number of terms.

### 3.1 Be 4 ^1^*S*

The Be 3 ^1^*S* wave function formed the basis for the Be 4 ^1^*S* expansion, except 7*s* and 7*p* outer *L* orbitals were added to reflect the more diffuse nature of 4 ^1^*S* versus 3 ^1^*S*. Consequently, the 40 term orbital optimization is for a 23,424 term expansion, and the final optimization is for an 86 block, 37,915 CSF wave function. Table A2 (Appendix) gives the overall picture of the 4 ^1^*S* energy convergence with a 99,874 term expansion using an *s, p, d, f r_i j_* STO basis for the CSFs. The optimized exponents for 3 ^1^*S* and 4 ^1^*S*^7^7 The optimized orbital exponents are tabulated further in Table 5.  are in line with invariant *K* and inner *L* shell *s* and *p* orbitals, except for an anomalous *L*_1_ case for which we have no explanation other than the energy for 3 ^1^*S* with respect to *L*_1_ turned out to be very flat. At this point, the 203 term expansion analogous to the 3 ^1^*S* expansion is about 0.037 *µ*Ha worse that the ECG result [19], and hence a seven digit result.

Note the increased importance of the *s_K_*_1_*s_K_*_1_ terms compared to 3 ^1^*S*. These terms were added to introduce additional *K*-shell correlation and hence serve to gauge how well the *K* shell is represented^8^8 The CI term was moved to the end of the expansion with the other *s_K_*_1_*s_K_*_1_ terms to facilitate this.. The increased importance of these terms suggested that further testing of these term types was in order (as CSFs 204-207 show) as well as re-examination of the CORE and final adjustments, which resulted in additional blocks being added to the 4 ^1^*S* expansion and our final 220 CSF, 99,874 term expansion. In addition to changes to the Be^+^(1*s*1*s*2*s*) core, the nature of the correlation changes as the outermost electron increases in energy (*e.g.* 3*s* ⇒ 4*s*, 4*s* ⇒ 5*s*, *etc.*). The implications with respect to the *r_i j_* factors are

• *r*_12_ correlates with the *K* shell and will always be important;
• *r*_34_ correlates with open shell electrons;
• *r*_13_ correlates with *K* shell and inner *L* shell electrons (in the Be^+^[1*s*1*s*2*s*] core); and
• *r*_14_ correlates with the outermost *L* shell electron and will be less and less important as the outermost electron gets further and further away from the nucleus.

Note how this is reflected in the additional CSFs 204-220, where there are no *r*_34_ or *r*_14_ terms that contributed enough to be included in the expansion. Term types (*psps*+*dpps*) (1+*r*_12_+*r*_13_) are much more important here than in Be 3 ^1^*S* and also are indicative of how the nature of the correlation changes as the outermost electron becomes more diffuse.

## 4 Results and Discussion

Table 3 shows that the current best Be 3 ^1^*S* 82,807 expansion result is 18 nHa below the ECG results of Stanke *et al.* [19] and of Pachucki [32], and within 18 nHa of the most recent, best calculation [21].

Similarly, the current best Be 4 ^1^*S* 99,874 expansion result is 14 nHa below the ECG result of Stanke *et al.* [19], and within 40 nHa of the most recent, best 4 ^1^*S* calculation [21]. This table includes the results for the 5 ^1^*S* and 6 ^1^*S* states, which will turn out to be within 46 nHa of the most recent, best 5 ^1^*S* result and 26 nHa better than the best previous calculation for the 6 ^1^*S* state. The Hy-CI restriction of one *r_i j_* per CSF has been previously discussed [3, 11, 12, 29], and it has been pointed out [11, 12] that the unlinked *r_i j_r_kl_* (no indices in common) term types first occur in the four-electron case, with *r*_12_*r*_34_ expected to be the most important. In the most recent Be [11] and Li^−^ [12] work, it was pointed out that one can reason that the *r*_12_*r*_34_ double cusp leads to two convergence problems, (*ss*)*_K_ r*_12_ × [*L*-shell cluster] and [*K*-shell cluster] × (*ss*)*_L_ r*_34_, when the double cusp is represented by a superposition of normal Hy-CI term types. The [*K*-shell cluster] and the [*L*-shell cluster] are both basically a linear combination of pair functions *ss* + *pp* + *dd* + *f f* + *gg* + *...*.

**Table 3:**
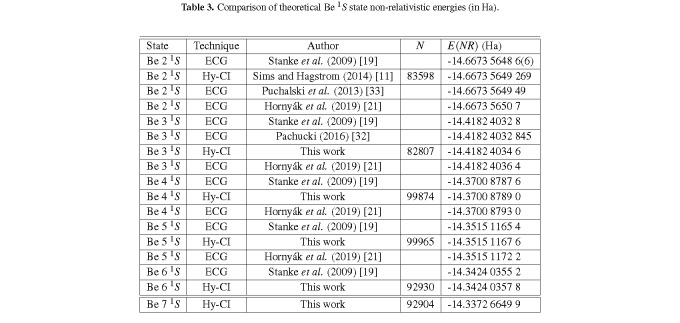
Comparison of theoretical Be ^1^*S* state non-relativistic energies (in Ha).

How well the convergence of the *r*_12_*r*_34_ term type (“double cusp problem”) is treated in Hy-CI for four-electron atoms and anions was analyzed in Table 3 of the 2011 work of Sims and Hagstrom [29] for Be and in Table 3 of the 2017 work by Sims [12] for Li^−^. The results suggested that (for the four-electron problem), compared to the slow, cusp-connected convergences in typical CI calculations, the convergence is fast and that this correlation type can be accurately, albeit slowly, represented within the Hy-CI model. Table 4 compiles the results from the expansions in Tables A1 and A2 of the Appendix, which explore this point for the first two excited states of Be of ^1^*S* symmetry.

**Table 4:**
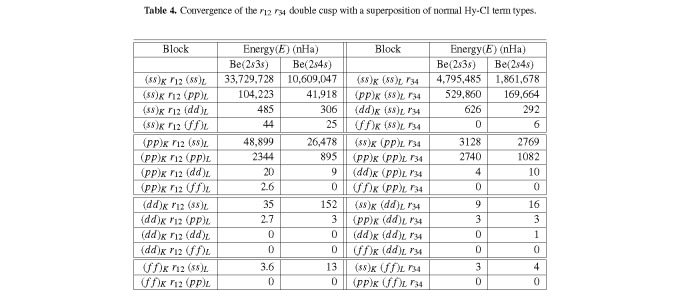
Convergence of the *r*_12_ *r*_34_ double cusp with a superposition of normal Hy-CI term types.

These results suggest that convergence is fast and that this correlation type can be accurately, albeit slowly, represented within the Hy-CI model for the more diffuse electronic distribution of Be(2*s*3*s*), as was the case for Li^−^, and that this trend continues for Be(2*s*4*s*) as well. Interestingly, the *K*-shell does not appear to be a problem, while the *L*-shell is not as converged in the *r*_12_ case. Addition of higher *l* STOs and blocks such as (*ss*)*_K_ r*_12_ (*gg*)*_L_*, (*ss*)*_K_ r*_12_ (*hh*)*_L_*, and (*ss*)*_K_ r*_12_ (*ii*)*_L_* could lead to perhaps an additional 5 nHa to 10 nHa.

Ultimately, slow convergence is built into Hy-CI for four or more electrons where one is trying to approximate the effect of the missing “double cusp” terms, as well as the *r_i j_r_ik_* terms first encountered with three electrons. Previous experience with Li suggests that the linked product terms *r_i j_r_ik_* should not be expected to give problems until the nanohartree level, but in going beyond the nanohartree level, the slow double cusp convergence would become even more of a problem. This suggests further study of the double cusp problem is necessary using codes explicitly designed for this (our code is not). The results do appear to support the conclusion in previous work, which stated that while 10-20 nHa accuracy can be achieved without any difficulty, doing substantially better will require greater flexibility in the atomic orbital basis, including adding (*ss*)*_K_ r*_12_ × [(*gg*)*_L_*, (*hh*)*_L_*, and possibly (*ii*)*_L_*] blocks, and extensive experimentation to find the best combination of CSFs and orbitals on which to base a much larger calculation.

### 4.1 Be 5^1^*S*

The Be 4 ^1^*S* wave function formed the basis for the Be 5 ^1^*S* expansion, and so the final optimization for 5 ^1^*S* is for the same 86 block, 37,915 CSF wave function as for 4 ^1^*S*.

Table A3 of the Appendix gives an overall picture of the 5 ^1^*S* energy convergence with a 99,965 term expansion using an *s, p, d, f r_i j_* STO basis for the CSFs. The optimized exponents for Be 5 ^1^*S*^9^9 The optimized orbital exponents are tabulated further on in Table 5. continue the 3 ^1^*S* and 4 ^1^*S* trend of invariant *K* and inner *L* shell *s* and *p* orbitals appropriate for a Be^+^(1*s*1*s*2*s*) core,^10^10 With the one anomalous Be 3 ^1^*S L*_1_ orbital exponent. whereas the *L*_2_ and *L*_4_ exponents here are for a Be(2*s*5*s*) excited state, so while the *L*_4_ *p* orbital exponent is the same as for Be(2*s*4*s*), the *L*_2_ orbital exponent is more diffuse than the *L*_2_ orbital exponent for Be(2*s*4*s*), and the *L*_2_ exponent for 3*s* ⇒ 5*s* is a reasonably smooth curve as it should be.

In the table, the 136 block, 62,123 term expansion is the same expansion (with different orbital exponents) as in the 99,874 term Be 4 ^1^*S* expansion. Blocks 137-142 are based on similar terms in the 4 ^1^*S* expansion but have been moved to immediately follow the CORE adjustments, since these terms are also “addbacks”, *i.e.*, additional energy obtained by expanding orbital basis sets via the difference tool. For the *psps* blocks, we note that the *r*_34_ and *r*_14_ blocks in general are not as important as the outermost electron moves further away from the nucleus, so these blocks remain as they are in 4 ^1^*S* but the (1+*r*_12_+*r*_13_) blocks are 7-7-7-7 instead of the 5-5-5-5 in 4 ^1^*S*. The *s_K_*_1_*s_K_*_1_ terms, added to introduce additional *K*-shell correlation and hence serve to gauge how well the *K* shell is represented, are here comparable to their importance in Be 3 ^1^*S* and of lesser importance than in Be 4 ^1^*S*. This drop in importance is more than offset by an increased importance of *dpps*(1+*r*_12_+*r*_13_) blocks, while {*psps*, *dsds*} (1+*r*_12_+*r*_13_) are also of increased importance compared to 3 ^1^*S* and 4 ^1^*S*, following the general pattern of correlation changes as the outer electron becomes more diffuse. The fact that {*psps*, *dsds*} (1+*r*_12_+*r*_13_) is of increased importance should not be surprising considering how important the {*psp*, *dsd*} x *R* blocks are in Li^11^11 See Table I of Ref. [34].. The result of the 99,965 term Be 5 ^1^*S* calculation is tabulated in Table 3 and turns out to be, as noted previously, within 46 nHa of the recent, best results of Ref. [21].

### 4.2 Be 6^1^S

The Be 5 ^1^S wave function formed the basis for the 6 ^1^S expansion, and so the final optimization for 6 ^1^*S* is for the same 86 block, 37,915 CSF wave function as for 5 ^1^*S*.

Table A4 of the Appendix gives an overall picture of the 6 ^1^*S* energy convergence with a 92,930 term expansion using an *s, p, d, f r_i j_* STO basis for the CSFs. The optimized exponents for 6 ^1^*S*^12^12 The optimized orbital exponents are tabulated further on in Table 5. continue the Be 3 ^1^*S*, 4 ^1^*S*, and 5 ^1^*S* trend of invariant *K* and inner *L* shell *s* and *p* orbitals appropriate for a Be^+^(1*s*1*s*2*s*) core,^13^13 With the one anomalous 3 ^1^*S L*_1_ orbital exponent. whereas the *L*_2_ and *L*_4_ exponents here are for a 6 ^1^*S* excited state, so while the L_4_ *p* orbital exponent is about the same as for 5 ^1^*S*, the L_2_ orbital exponent is more diffuse than the L_2_ orbital exponent for 5 ^1^*S*, and the L_2_ exponent for 3s⇒ 6s is a reasonably smooth curve, as it should be.

The fundamental difference between this expansion and the Be 5 ^1^*S* expansion is that, in all terms, the outer *L* shell electron basis in each block includes an *n* = 6 orbital since we are dealing with a 6 ^1^*S* state. So, for example, the *spsp* 1:5*s_K_* 2:5*p_L_*_1_ 1:5*s_L_*_1_ 2:5*p_L_*_4_ *R* becomes 1:5*s_K_* 2:5*p_L_*_1_ 1:5*s_L_*_1_ 2:6*p_L_*_4_ *R*, where an additional 6 *p_L_*_4_ orbital has been added to the *p_L_*_4_ set. With this exception, the blocks up to the addback blocks starting at block 113 are the same as in the 5 ^1^*S* expansion. In the addback section, for the *pppp* blocks, the *r*_13_ and *r*_14_ blocks drop out. For the final adjustments, the *dsds* blocks are too small to be included, and the outermost *L* shell electron has gotten far enough away from the nucleus that the *r*_34_ and *r*_14_ blocks no longer need to be included.

The 6 ^1^*S* wave function at this point followed almost directly from the 5 ^1^*S* wave function, with the expansion of the outer *L* shell electron basis in each block to include an *n* = 6 orbital where needed. Again, we see an increased importance for the {*dpps*, *psps*, and *dsds*} (1+*r*_12_+*r*_13_) blocks compared to Be(2*sns*), *n<* 6. The result of a 92,930 term calculation is tabulated in Table 4 and turns out to be, as noted previously, 26 nHa lower than the results of Stanke et al. [19].

### 4.3 Be 7^1^*S*

The Be 6 ^1^*S* wave function formed the basis for the 7 ^1^*S* expansion, but now in addition to making sure that the outermost orbital basis set includes an *n* = 7 orbital, the CORE blocks are expanded to include an additional *L* shell orbital for both the inner and outer shell electrons. Hence, the size of the 86 block, 37,915 CSF wave function used in optimizing the 6 ^1^*S* wave function is now 52,294 CSFs, and it is this wave function that is used for the optimization of the 7 ^1^*S* basis orbitals.

Table A5 of the Appendix gives an overall picture of the Be 7 ^1^*S* energy convergence with a 92,904 term expansion using an *s, p, d, f r_i j_* STO basis for the CSFs. The optimized exponents for Be 7^1^*S* continue the 3 ^1^*S*, 4 ^1^*S*, 5 ^1^*S*, and 6 ^1^*S* trend of invariant *K* and inner *L* shell *s* and *p* orbitals appropriate for a Be^+^(1*s*1*s*2*s*) core,^14^14 With the one anomalous Be 3 ^1^*S L*_1_ orbital exponent. whereas the L_2_ and L_4_ exponents here are for a 7 ^1^*S* excited state, so while the *L*_4_ *p* orbital exponent is about the same as for 6 ^1^*S*, the *L*_2_ orbital exponent is more diffuse than the *L*_2_ orbital exponent for 6 ^1^*S*, and the *L*_2_ exponent for 3*s* ⇒ 7*s* is a reasonably smooth curve, as it should be.

The fundamental difference between this expansion and the Be 6 ^1^*S* expansion is that, in all terms, the outer *L* shell electron basis in each block includes an *n* = 7 orbital, since we are dealing with a 7 ^1^*S* state. So, for example, the *spsp* 1:5*s_K_* 2:5*p_L_*_1_ 1:5*s_L_*_1_ 2:6*p_L_*_4_ *R* becomes 1:5*s_K_* 2:5*p_L_*_1_ 1:5*s_L_*_1_ 2:7*p_L_*_4_ *R*, where an additional 7 *p_L_*_4_ orbital has been added to the *p_L_*_4_ set. Since the CORE blocks now contain an outermost *L n*= 8 orbital, many of the addback blocks are superfluous and are not included in the expansion. Except for these differences, the 7 ^1^*S* expansion looks very much like the 6 ^1^*S* expansion, with a few less important blocks having dropped out.

## 5 Conclusion

Table 5 is a table of the optimum zeta values for each of the species explored here.

**Table 5:**
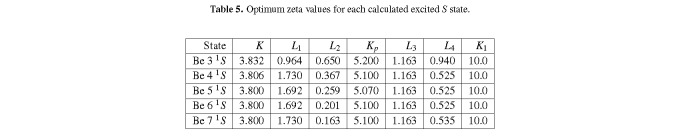
Optimum zeta values for each calculated excited *S* state.

Here, *K*, *K_p_*, *L*_1_, *L*_3_, and *K*_1_ are basically fixed after the 3 ^1^*S* state, because the Be^+^(1*s*1*s*2*s*) core changes very little as one approaches ionization and the outer *L* shell orbital gets further and further away from the nucleus. *L*_2_ should vary smoothly as the outer electron gets further and further away from the nucleus, and indeed it does. *L*_4_ should do the same to a lesser extent, but we find that it varies very little in fact.

The best non-relativistic energies for Be 3 ^1^*S*, 4 ^1^*S*, 5 ^1^*S*, and 6 ^1^*S* obtained are -14.4182 4034 638 Ha,-14.3700 8789 0 Ha, -14.3515 1167 6 Ha, and -14.3424 0357 8 Ha, respectively. In addition, the best non-relativistic energy for Be 7 ^1^*S* obtained is -14.3372 6649 96 Ha. The 6 ^1^*S* result is superior to the known reference energy for that state, while for the 7 ^1^*S* state, there is no other comparable calculation.

We have previously shown that Hy-CI is capable of high-precision results for not only ground states, but excited states as well, for three-electron atoms, specifically the Li atom. Hy-CI has from its inception been an attempt to extend the success of the Hy method to systems with more than three electrons. A fundamental feature in the method is the restriction of one *r_i j_* per CSF, which has achieved 10-20 nHa accuracy for the ground states of the Be isoelectronic series with *Z* ≥ 4. For both extension of the method to *N >* 4 as well as for excited states of four-electron systems, how well the method handles the “double cusp” problem when the *L* shell is more diffuse than in the ground state needs to be studied. This work continues the work begun with Li^−^ (which turns out to have a Be(2*s*3*s*)-like structure as calculations have shown down to the block level [12]) by examining how well Hy-CI can treat states successively more diffuse than the ground state.

This is accomplished by examining successively higher states of Be of ^1^*S* symmetry. Specifically, a study of the Be(2*sns*) excited states, *n* = 3,7 has been carried out. Compared to Be, this is a more diffuse system, one in which a weaker “double cusp” would be expected, which should make Hy-CI more favorable, but also more CI-like, so some of the advantage of Hy-CI is lost, perhaps. Indeed, we find that *r*_14_, which correlates the outermost *L* shell electron and to a lesser extent, *r*_34_, which correlates open shell electrons, become less and less important as the outer *L* shell electron becomes more distant, and this compensates for blocks in which the basis has been expanded to represent the more diffuse charge distribution. Based on the calculations reported in Sec. III and Sec. IV here, one can conclude that this correlation type can be accurately represented for the more diffuse distribution of Be excited states of ^1^*S* up through 7 ^1^*S*. Combined with the previous Be isoelectronic series results, these calculations exemplify the level of accuracy that is now possible with Hy-CI in describing not only the ground state of Li-, Be and Be-like ions, but also excited states as well.

## 6 Appendix

Tables A1-A5 list the final Hy-CI expansions for the 3 ^1^*S*, 4 ^1^*S*, 5 ^1^*S*, 6 ^1^*S*, and 7 ^1^*S* states, respectively. In the tables, ∆*E* for each CSF block is the amount by which that block lowers the non-relativistic energy when added to the expansion. Column 1 lists the CSF block specifications used to generate the CSF terms for the various block types, in the order electron 1 (*α* spin), electron 2 (*β* spin), electron 3 (*α* spin), electron 4 (*β* spin). For example, in the first line 1:9*s_K_* for the first electron means the basis orbitals are 1*s_K_* through 9*s_K_* orbitals (where *K* indicates an orbital exponent appropriate for a *K* shell electron). All of the listed basis orbitals are used to generate all of the CSFs that are unique for this basis set selection, except that *N_max_*, the sum of the powers of *r* in the Hartree product (HP), must be less than or equal to 16 unless an explicit *r*-sum is specified. The choice of terms is highly regular, there having been no attempt to cut down on the number of terms. The number of unique terms (CSFs) in a block can be computed from the listed basis orbitals and the condition that *N_max_* ≤ 16 (or ≤ the explicitly specified *N_max_*). For example, consider 1:6*s_K_*_1_ 1:6*s_K_*_1_ 1:5*s_L_*_1_ 1:5 *s_L_*_2_ *r*_34_ in the last row of Table A1. There are 6 x 7 / 2 = 21 unique pairs of orbitals for electrons 1 and 2 and 5 x 5 = 25 unique pairs of orbitals for electrons 3 and 4. Since the *K* shell orbital exponent is different from the *L* shell orbital, there are (21 x 25) = 525 different CSF terms for this block. Applying the condition that the *r*-sum, the sum of powers of *r* for the four orbitals in a term, has to be ≤ 16, the number of terms for this CSF block is reduced to 521 (see Ref. [29] for further details).

**Table A1:**
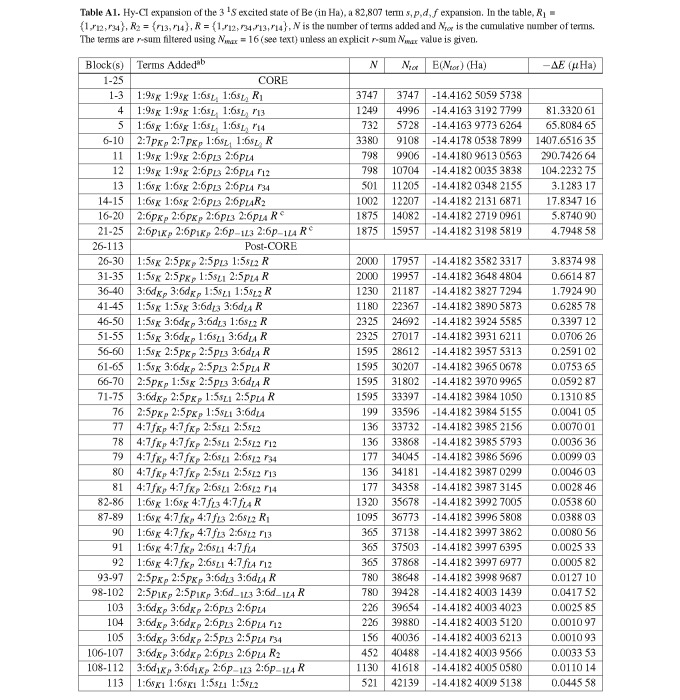

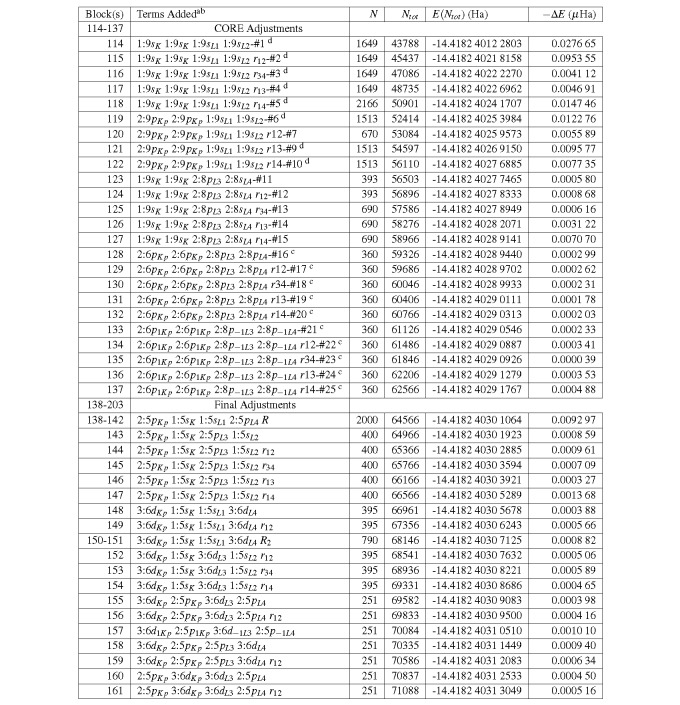

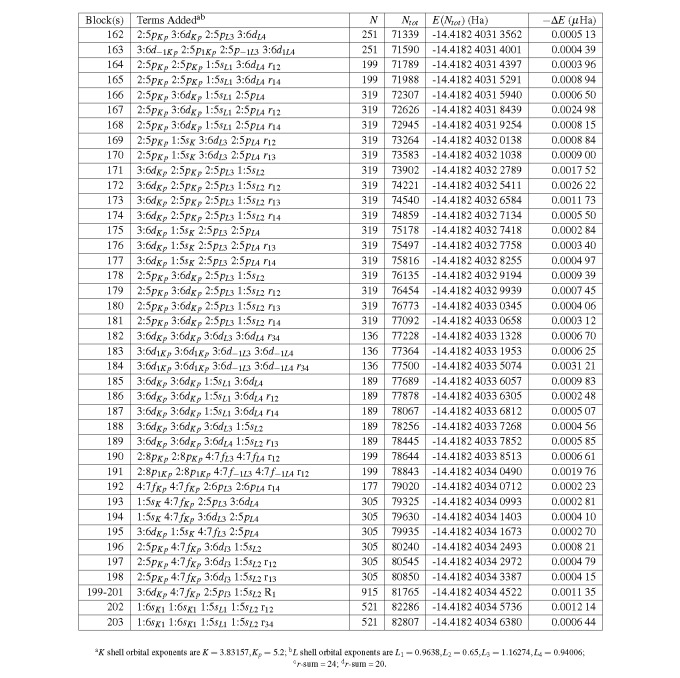
Hy-CI expansion of the 3 ^1^*S *excited state of Be (in Ha), a 82,807 term *s, p, d, f *expansion. In the table, *R*_1_ =*{*1,*r*_12_*, r*_34_*}*, *R*_2 _= *{r*_13_*, r*_14_*}*, *R *= *{*1,*r*_12_*, r*_34_,*r*_13_*, r*_14_*}*, *N *is the number of terms added and *N_tot _*is the cumulative number of terms. The terms are *r*-sum filtered using *N_max _*= 16 (see text) unless an explicit *r*-sum *N_max _*value is given.

**Table A2:**
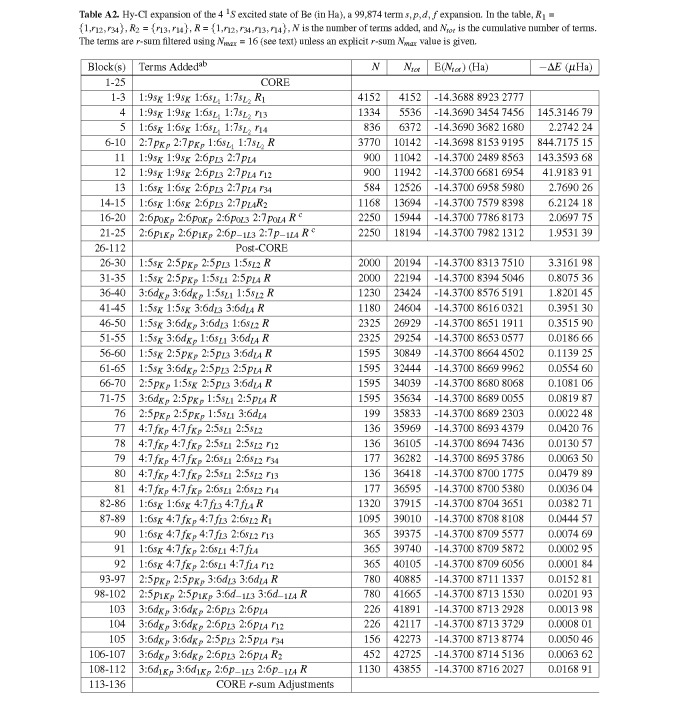

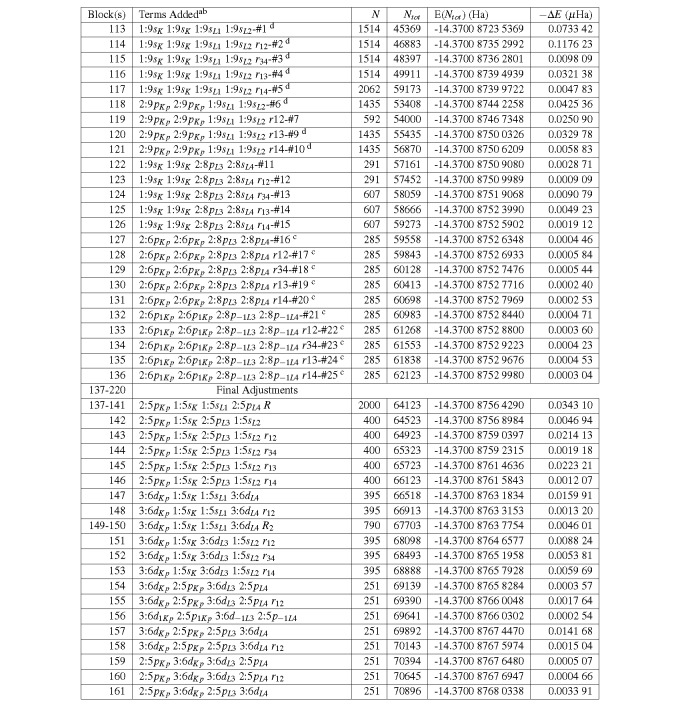

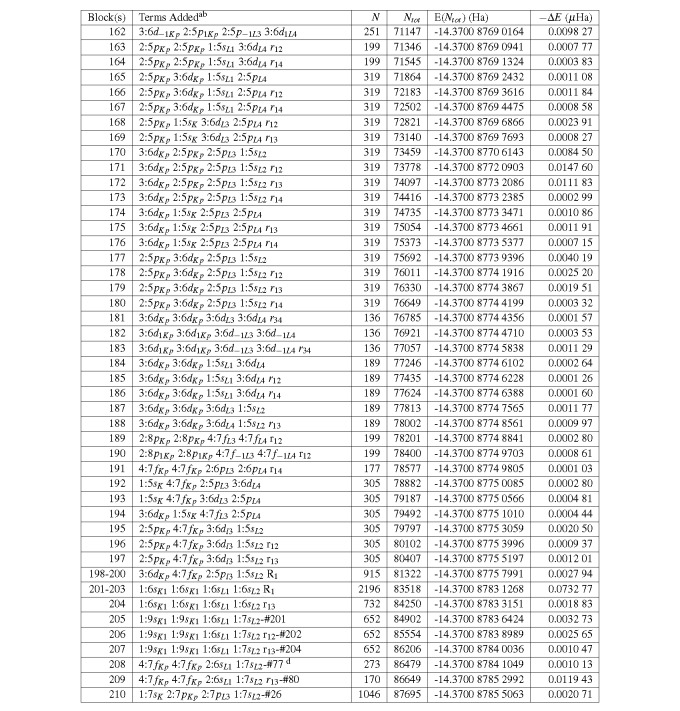

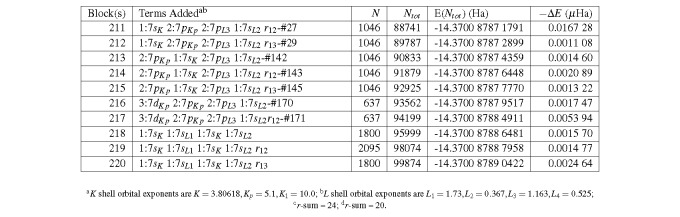
Hy-CI expansion of the 4 ^1^*S *excited state of Be (in Ha), a 99,874 term *s, p, d, f *expansion. In the table, *R*_1_ =*{*1,*r*_12_*, r*_34_*}*, *R*_2 _= *{r*_13_*, r*_14_*}*, *R *= *{*1,*r*_12_*, r*_34_,*r*_13_*, r*_14_*}*, *N *is the number of terms added, and *N_tot _*is the cumulative number of terms. The terms are *r*-sum filtered using *N_max _*= 16 (see text) unless an explicit *r*-sum *N_max _*value is given.

**Table A3:**
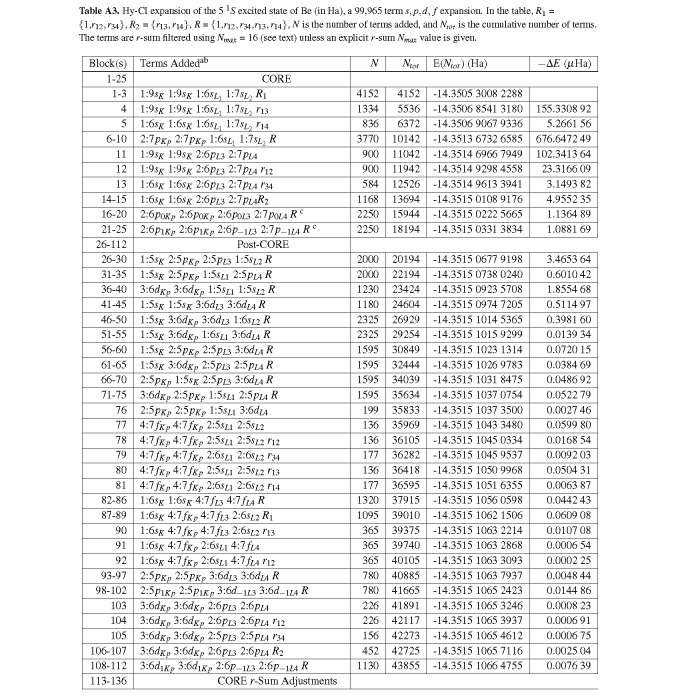

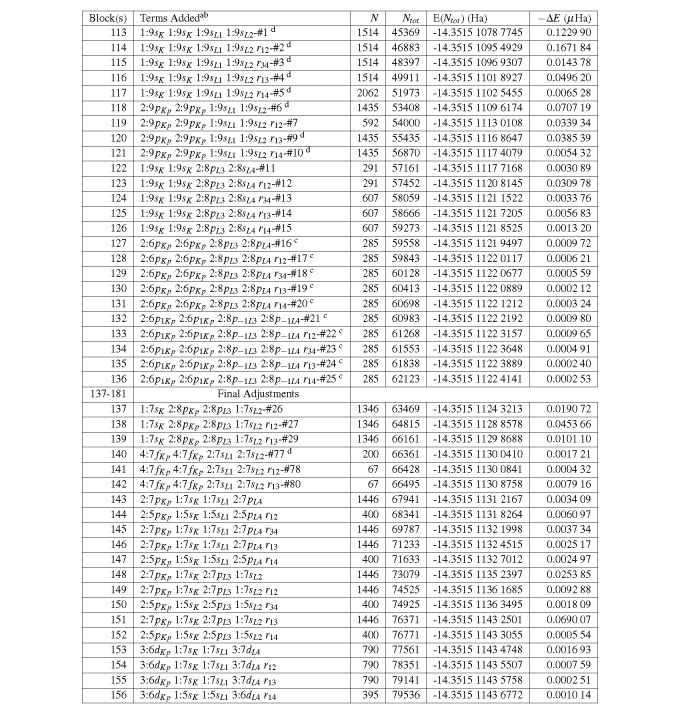

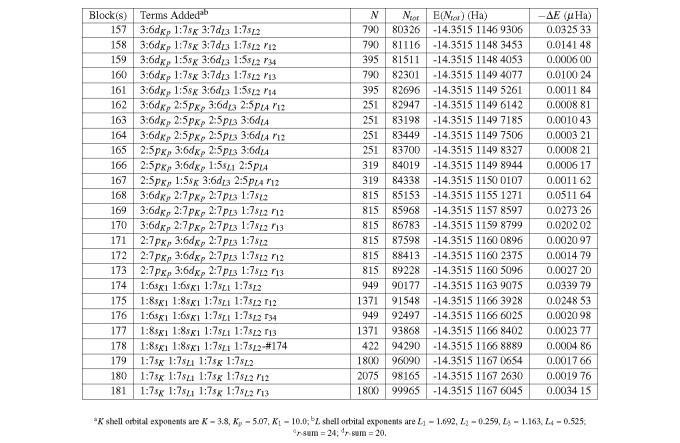
Hy-CI expansion of the 5 ^1^*S *excited state of Be (in Ha), a 99,965 term *s, p, d, f *expansion. In the table, *R*_1_ = *{*1,*r*_12_*, r*_34_*}*, *R*_2 _= *{r*_13_*, r*_14_*}*, *R *= *{*1,*r*_12_*, r*_34_,*r*_13_*, r*_14_*}*, *N *is the number of terms added, and *N_tot _*is the cumulative number of terms. The terms are *r*-sum filtered using *N_max _*= 16 (see text) unless an explicit *r*-sum *N_max _*value is given.

**Table A4:**
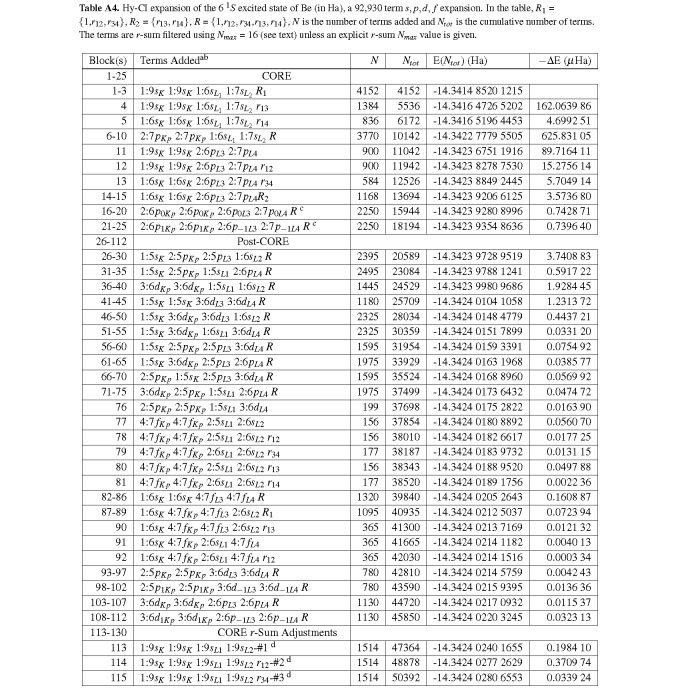

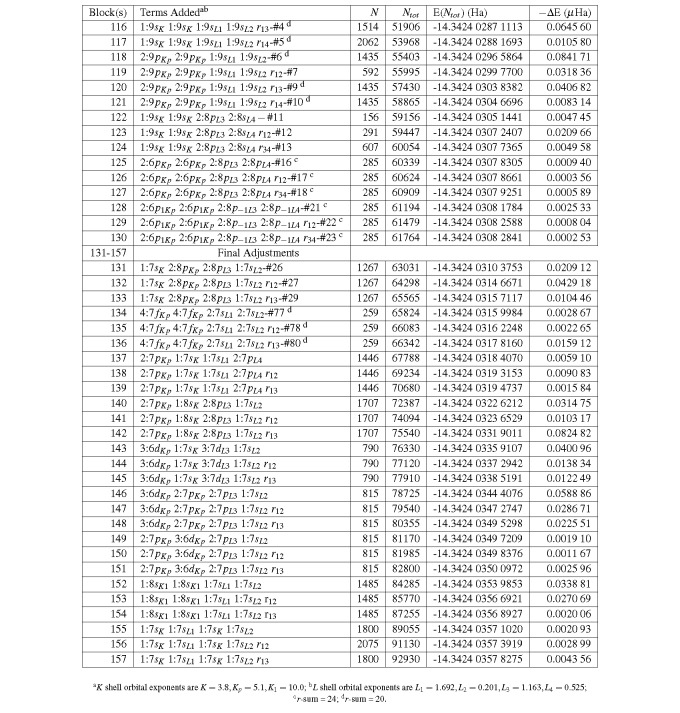
Hy-CI expansion of the 6 ^1^*S *excited state of Be (in Ha), a 92,930 term *s, p, d, f *expansion. In the table, *R*_1_ = *{*1,*r*_12_*, r*_34_*}*, *R*_2 _= *{r*_13_*, r*_14_*}*, *R *= *{*1,*r*_12_*, r*_34_,*r*_13_*, r*_14_*}*, *N *is the number of terms added and *N_tot _*is the cumulative number of terms. The terms are *r*-sum filtered using *N_max _*= 16 (see text) unless an explicit *r*-sum *N_max _*value is given.

**Table A5:**
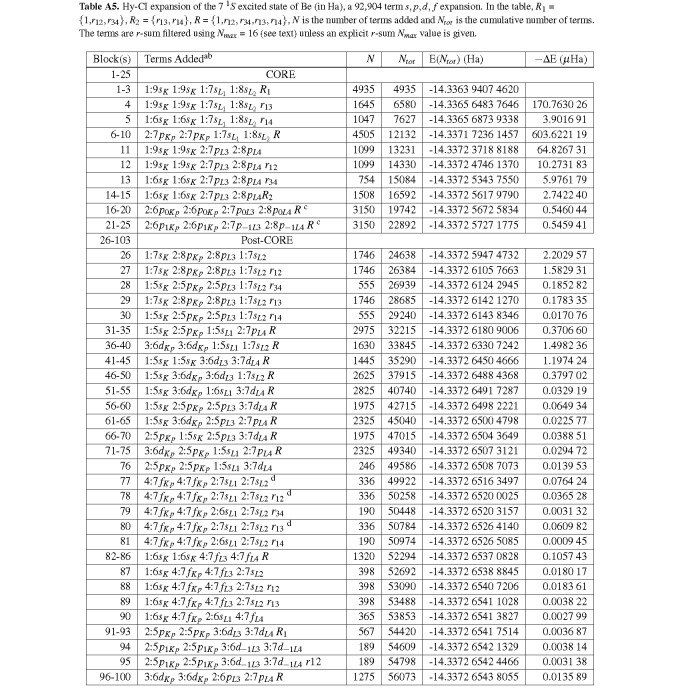

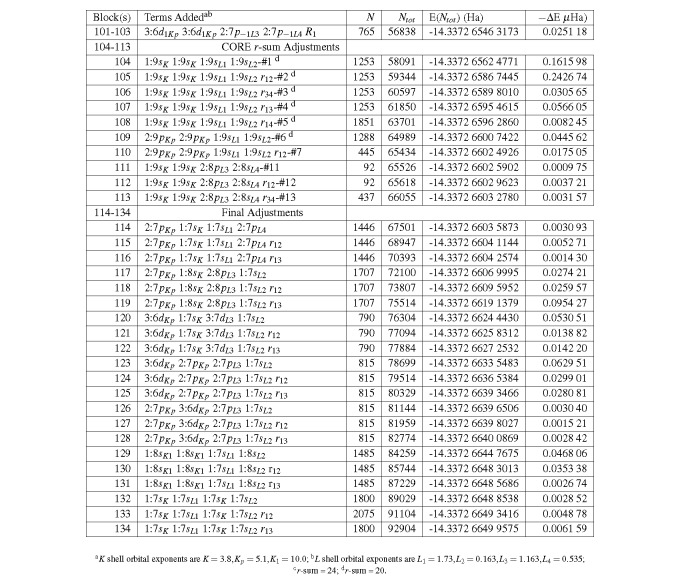
Hy-CI expansion of the 7 ^1^*S *excited state of Be (in Ha), a 92,904 term *s, p, d, f *expansion. In the table, *R*_1_ = *{*1,*r*_12_*, r*_34_*}*, *R*_2 _= *{r*_13_*, r*_14_*}*, *R *= *{*1,*r*_12_*, r*_34_,*r*_13_*, r*_14_*}*, *N *is the number of terms added and *N_tot _*is the cumulative number of terms. The terms are *r*-sum filtered using *N_max _*= 16 (see text) unless an explicit *r*-sum *N_max _*value is given.

## References

[1] Rychlewski J (ed) (2003) Explicitly Correlated Wave Functions in Chemistry and Physics Theory and Applications (Kluwer Academic Publishers, Dordrecht, Netherlands) 1st Ed.

[2] Hylleraas EA (1929) Neue Berechnung der Energie des Heliums in Grundzustande, sowie des tiefsten Terms von Orthohelium. Zeitschrift fur Physik 54:347–366. 10.1007/BF01375457

[3] Sims JS, Hagstrom SA (1971) Combined configuration interaction - Hylleraas type wave function study of the ground state of the beryllium atom. Physical Review A 4(3):908–916. 10.1103/PhysRevA.4.908

[4] Woznicki W (1971) On the method of constructing the variational wave function for many-electron systems. Theory of electronic shells in atoms and molecules, ed Jucys A (Mintis, Vilnius, Lithuania) 1st Ed., p 103.

[5] Handy NC (1973) Towards an understanding of the form of correlated wavefunctions for atoms. Journal of Chemical Physics 58:279–287. 10.1063/1.1678918

[6] Kutzelnigg W (1985) *r*_12_-Dependent terms in the wave function as closed sums of partial wave amplitudes for large *l.* Theoretica Chemica Acta 68:445–469. 10.1007/BF00527669

[7] Kong L, Bischoff F, Valeev EF (2011) Explicitly correlated r12/f12 methods for electronic structure. Theoretica Chemica Acta 112:75–107. 10.1021/cr200204r 22176553

[8] Ruiz MB (2015) Hylleraas-Configuration interaction calculations on the 1^1^*S* ground state of the helium atom. Journal of Coordination Chemistry 68(17–18):3340–3361. 10.1080/00958972.2015.1064907

[9] Nakatsuji H, Nakashima H, Kurokawa YI (2018) Solving the Schro¨dinger equation of atoms and molecules: Chemical formula theory, free-complement chemical-formula theory, and intermediate variational theory. Journal of Chemical Physics 149:114105. 10.1063/1.5040376 30243277

[10] Nakatsuji H, Nakashima H (2019) Solving the Schro¨dinger equation of atoms and molecules: Variational study of the ground and excited states of Be and Li atoms. Journal of Chemical Physics 150:044105. 10.1063/1.5065565 30709316

[11] Sims JS, Hagstrom SA (2014) Hylleraas-configuration-interaction nonrelativistic energies for the ^1^*S* ground states of the beryllium isoelectronic sequence. Journal of Chemical Physics 140:224312. 10.1063/1.4881639 24929393

[12] Sims JS (2017) Hylleraas-configuration interaction study of the ^1^*S* ground state of the negative Li ion. Journal of Physics B: Atomic, Molecular, and Optical Physics 50:245003. 10.1088/1361-6455/aa961e PMC5785787 29379225

[13] King FW (1997) Progress on high-precision calculations for the ground state of atomic lithium. Journal of Molecular Structure (Theochem) 400:7–56. 10.1016/S0166-1280(97)90265-7

[14] Pachucki K, Komasa J (2006) Electron affinity of Li. Journal of Chemical Physics 125:204304. 10.1063/1.2393226 17144697

[15] Stanke M, Kedziera D, Bubin S, Adamowicz L (2007) Electron affinity of ^7^*Li* calculated with the inclusion of nuclear motion and relativistic corrections. Journal of Chemical Physics 127:134107. 10.1063/1.2755767 17919011

[16] Bubin S, Komasa J, Stanke M, Adamowicz L (2009) Isotope shift in the electron affinity of lithium. Journal of Chemical Physics 131:234112. 10.1063/1.3275804 20025319

[17] Hussein A (2019) CI calculations for the ground and lowest core-excited states of Li and Li^−^. Physica B: Condensed Matter 570:66–72. 10.1016/j.physb.2019.06.006

[18] King FW, Quicker D, Langer J (2011) Compact wave functions for the beryllium isoelectronic series, Li^−^ to Ne^+6^: A standard Hylleraas approach. Journal of Chemical Physics 134:124114. 10.1063/1.3569565 21456652

[19] Stanke M, Komasa J, Bubin S, Adamowicz L (2009) Five lowest ^1^S states of the Be atom calculated with a finite-nuclear-mass approach and with relativistic and QED corrections. Physical Review A 80:022514. 10.1103/PhysRevA.80.022514 20331283

[20] Stanke M, Bubin S, Adamowicz L (2019) Lowest ten singlet P states of beryllium calculated with all-electron explicitly correlated Gaussian functions. Journal of Physics B: Atomic, Molecular, and Optical Physics 52:155002. 10.1088/1361-6455/ab2510

[21] Hornyak I, Adamowicz L, Bubin S (2019) Lowest excitation energy of ^9^Be. Physical Review A 100:032504. 10.1103/PhysRevA.100.032504 17678358

[22] Lowdin PO (1964) Angular momentum wavefunctions constructed by projector operators. Reviews of Modern Physics 36:966–976. 10.1103/RevModPhys.36.966

[23] Condon EU, Shortley GH (1963) The Theory of Atomic Spectra (Cambridge University Press, Cambridge, UK) 1st Ed.

[24] Cencek W, Rychlewski J (1993) Many-electron explicitly correlated Gaussian functions. I. General theory and test results. Journal of Chemical Physics 98(2):1252–1261. 10.1063/1.464293

[25] Golub GH, Van Loan CF (2013) Matrix Computations (Johns Hopkins University Press, Baltimore, MD) 4th Ed.

[26] Ralston A, Rabinowitz P (1978) A First Course in Numerical Analysis (McGraw-Hill, New York) 2nd Ed.

[27] Meyer CD (2000) Matrix Analysis and Applied Linear Algebra (SIAM, Philadelphia, PA) 1st Ed.

[28] Message Passing Interface Forum (1994) MPI: A Message-Passing Interface Standard. International Journal of Supercomputer Applications 8(3/4):159–416.

[29] Sims JS, Hagstrom SA (2011) Hylleraas-configuration interaction study of the ^1^*S* ground state of neutral beryllium. Physical Review A 83:032518. 10.1103/PhysRevA.83.032518

[30] Sims JS, Hagstrom SA (1971) One center *r_i j_* integrals over Slater-type orbitals. Journal of Chemical Physics 55(10):4699–4710. 10.1063/1.1675567

[31] Stanke M, Kedziera D, Bubin S, Adamowicz L (2007) Lowest excitation energy of ^9^Be. Physical Review Letters 99:043001. 10.1103/PhysRevLett.99.043001 17678358

[32] Pachucki K (2016). Personal communication.

[33] Puchalski M, Komasa J, Pachucki K (2013) Testing quantum electrodynamics in the lowest singlet states of the beryllium atom. Physical Review A 87:030502. 10.1103/PhysRevA.87.030502

[34] Sims JS, Hagstrom SA (2009) Hy-CI variational calculations for the 2 ^2^*S* ground state of neutral lithium and the first five excited ^2^*S* states. Physical Review A 80(5):052507. 10.1103/PhysRevA.80.052507

